# Antimicrobial Resistance in Bacteria from Meat and Meat Products: A One Health Perspective

**DOI:** 10.3390/microorganisms11102581

**Published:** 2023-10-17

**Authors:** Sara Conceição, Maria Cristina Queiroga, Marta Laranjo

**Affiliations:** 1 MED—Mediterranean Institute for Agriculture, Environment and Development & CHANGE—Global Change and Sustainability Institute, Institute for Advanced Studies and Research, Universidade de Évora, Pólo da Mitra, Ap. 94, 7006-554 Évora, Portugal; smpc@uevora.pt (S.C.); crique@uevora.pt (M.C.Q.); 2 Departamento de Medicina Veterinária, Escola de Ciências e Tecnologia, Universidade de Évora, Pólo da Mitra, Ap. 94, 7006-554 Évora, Portugal

**Keywords:** antimicrobial resistance, One Health, multidrug-resistant bacteria, food safety, farm-to-fork transmission

## Abstract

According to the 2030 Agenda of the United Nations, one of the sustainable development goals is to ensure sustainable consumption and production patterns. The need to ensure food safety includes, other than microbiological hazards, concerns with antimicrobial-resistant (AMR) bacteria. The emergence of resistant bacteria in the food industry is essentially due to the abusive, and sometimes incorrect, administration of antimicrobials. Although not allowed in Europe, antimicrobials are often administered to promote animal growth. Each time antimicrobials are used, a selective pressure is applied to AMR bacteria. Moreover, AMR genes can be transmitted to humans through the consumption of meat-harbouring-resistant bacteria, which highlights the One Health dimension of antimicrobial resistance. Furthermore, the appropriate use of antimicrobials to ensure efficacy and the best possible outcome for the treatment of infections is regulated through the recommendations of antimicrobial stewardship. The present manuscript aims to give the current state of the art about the transmission of AMR bacteria, particularly methicillin-resistant *S. aureus*, ESBL-producing Enterobacteriaceae, and vancomycin-resistant *Enterococcus* spp., along with other ESKAPE bacteria, from animals to humans through the consumption of meat and meat products, with emphasis on pork meat and pork meat products, which are considered the most consumed worldwide.

## 1. Introduction

Over the last few decades, an increase in antimicrobial-resistant (AMR) bacteria has been observed, including resistance to antimicrobials not authorized in veterinary medicine, which have been reported in meat products [1]. More recently, several policies have been designed to reduce AMR. New therapeutic strategies have been developed, such as the use of bacteriophages, antimicrobial peptides, and phytobiotics such as essential oils or propolis ethanol extracts [2,3,4,5,6,7,8,9]. In addition, the “Antimicrobial Stewardship” strategy was developed in 2007 to obtain better clinical outcomes for the treatment of infections involving a careful selection of antimicrobials, as well as their route, dose, and treatment duration [10]. Furthermore, One Health aims to achieve optimal human health and well-being while also being internally related to animal health and the environment. One Health promotes the fight against antimicrobial resistance because both humans and animals are affected by the same bacteria and are also treated with the same antimicrobials. The One Health approach is considered a collaboration between different sectors, developed in 2003 by the now joint quadripartite consortium, including the Food and Agriculture Organisation of the United Nations (FAO), the World Health Organisation (WHO), the World Organisation for Animal Health (WOAH, founded as OIE), and the United Nations Environment Programme (UNEP) [11]. Moreover, in September 2015, the United Nations developed a global action plan with 193 countries. This action plan, named “Transforming Our World: The 2030 Agenda for Sustainable Development”, has sustainable development as its main goal and has 17 Sustainable Development Goals (SDGs) [12,13,14]. For these SDGs to be fulfilled, around 170 targets were established to address several issues. Examples of these issues are climate change, environmental degradation, and social inequality [14]. However, SDGs are also related to food, namely SDG 12, which aims to promote responsible and sustainable food production and consumption [15]. Thus, SGD 12 is directly linked to one of the current problems regarding food, the antimicrobial-resistant bacteria present in food.

Antimicrobial resistance is considered a complex problem and a global health concern for both humans and animals. Around 2.8 million human cases of infections by antimicrobial-resistant bacteria and 700,000 deaths are reported annually, and this number could reach 10 million by 2050 if AMR is not reduced [16,17]. Antimicrobials have been used extensively and without respecting the therapy guidelines, mostly in low- and middle-income countries [18]. Resistant bacteria have been found in humans, animals, food, and the environment, leading to the transmission of resistance genes across bacterial species and between animals, humans, and the environment [18,19,20]. Bacteria isolated from food-producing animals have shown high AMR for most antimicrobials used in human medicine. About 54% of *Escherichia coli* and *Klebsiella* spp. showed high resistance to third-generation cephalosporine [16,18]. The existence of AMR bacteria in foods is mainly due to the excessive use of antimicrobials in food-producing animals and, consequently, the transmission of the AMR bacteria to humans through consumption (also known as “farm-to-fork” transmission) [21,22].

The WHO reported high levels of bacterial AMR worldwide, highlighting the need for a One Health approach to deal with the AMR crisis. The One Health approach works at a local, national, and global level, with the collaboration of policymakers, stakeholders, practitioners, and researchers [18,23].

There are three microbial groups in food products for which AMR can be considered a problem: *Staphylococcus* spp. (particularly, methicillin-resistant *S. aureus*), extended-spectrum beta-lactamase (ESBL)-producing *Enterobacteriaceae*, and some lactic acid bacteria (mainly, vancomycin-resistant *Enterococcus* spp.) [24,25,26]. Currently, the most threatening multidrug-resistant (MDR) bacteria belong to the ESKAPE group, characterized by the ability to escape the action of various classes of antimicrobials, whether in humans or animals. ESKAPE bacteria comprise *Enterococcus* (E.) *faecium*, *Staphylococcus* (S.) *aureus*, *Klebsiella* (K.) *pneumoniae*, *Acinetobacter* (A.) *baumannii*, *Pseudomonas* (P.) *aeruginosa*, and *Enterobacter* (E.) species [2,22,27].

The present review aims to analyse the state of the art related to the AMR in various types of meat and meat products, with an emphasis on pork meat and pork meat products, which are considered the most consumed worldwide. In this type of food, there are three microbiota groups of interest, namely methicillin-resistant *S. aureus*, ESBL-producing *Enterobacteriaceae*, and vancomycin-resistant *Enterococcus* spp. Moreover, ESKAPE bacteria, which include bacteria from the three abovementioned groups, are also addressed as they have the highest impact on AMR bacteria in the food industry [28,29,30,31].

## 2. The One Health Approach

Antimicrobials have dramatically improved human and animal health. However, the effectiveness of antimicrobials has decreased over the years, giving rise to resistant bacterial strains. Due to the excessive use of antimicrobials in hospitals, in the community, and the livestock sector, there was an emergence of MDR microorganisms. This led to a global AMR increase and a threat to public health as the existence of AMR bacteria hampers the treatment of diseases. Additionally, resistant bacteria may enter the food chain through consumption, increasing the risk of AMR in food pathogens [32,33]. Without effective antimicrobials, healthcare costs, disease occurrence, and mortality rates highly increase. The One Health approach is an integrated and unifying strategy towards the sustainable health of ecosystems, humans, and animals [34,35]. To fight the increase in AMR, the One Health approach establishes communication channels between different sectors for the development and implementation of AMR surveillance programs, achieving optimal health for humans, animals, and ecosystems [19,36].

Additionally, the One Health approach to antimicrobial stewardship is an ecological concept, and its main purpose is to improve prescribing practices by doctors and veterinarians [23,37]. To fulfil the purpose of One Health, there must be a surveillance of AMR transmission elements considered relevant to define the AMR transmission process between humans, animals, plants, and the environment [37].

Specifically concerning the pig industry, the surveillance must focus on the whole value chain, namely the production, slaughtering, and processing steps. Mitigation measures may include hygiene measures throughout the whole chain but also monitoring strategies, promoting both the use of biosafety methods and vaccine research and development [38]. Lately, innate immunomodulation is a new strategy that is currently being studied, where the innate immune memory is achieved through the stimulation of innate immune cells with non-related stimuli [39]. This phenomenon has been recently reported in pigs [40].

One Health, however, is a multi-hierarchical system; there is a problem with predictions, namely how a change in a particular level of the hierarchy affects the remaining levels. Computer science managed to solve this problem with membrane computing modelling, which was recently applied to AMR prediction [37,41]. Through the One Health approach, it will be possible to develop new biochemical, microbiological, ecological, computational, and bioinformatics techniques, which will be necessary to understand and, also, to control the problem of AMR globally [37,42,43].

## 3. Antimicrobial Activity

Antimicrobials are natural, semi-synthetic, or synthetic compounds capable of killing bacteria or preventing bacterial growth. These are used in the treatment of bacterial infections in humans and animals, or as feed additives or synthetic growth promoters in animals and aquaculture [33].

**Figure 1:**
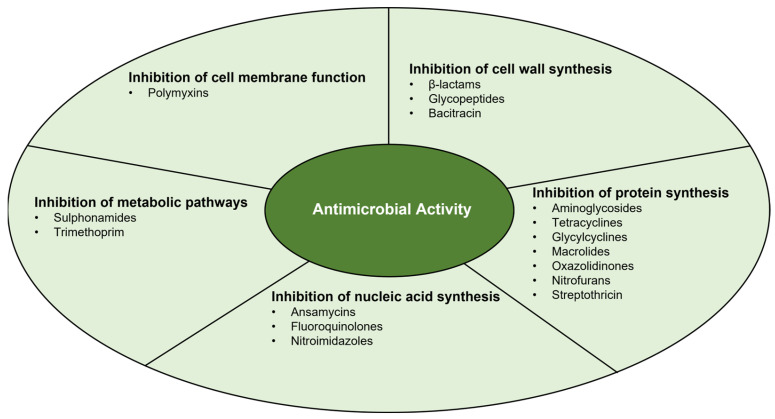
Main mechanisms of antimicrobial activity.

Antimicrobial activity may be divided into five main mechanisms, which are summarised in Figure 1 [44,45].

### 3.1. Inhibition of Cell Wall Synthesis

The bacterial cell wall is composed of peptidoglycan that generates mechanical support and allows the bacteria to survive under extreme situations (e.g., osmotic pressure changes) [44,46]. Peptidoglycan is a polymer formed by chains of glycans, formed by disaccharide subunits of N-acetylglucosamine and acetylmuramic acid, cross-linked by pentapeptide chains [44]. This component can be found in both Gram-negative and Gram-positive bacteria. In Gram-negative bacteria, the cell wall comprises 1 or 2 layers of peptidoglycan, while in Gram-positive bacteria, 10–40 layers are present [44,46].

There are different antimicrobials whose mechanisms of action inhibit cell wall synthesis: β-lactams, glycopeptides, and bacitracin, which is a polypeptide antibiotic [44,46,47]. Beta-lactams bind to transpeptidases (also called PBPs—penicillin-binding proteins), inhibiting the formation of peptide bonds between tetrapeptides that crosslink glycan chains, inactivating the PBPs, which results in the lysis of microorganisms [44,46]. Glycopeptides block cell wall synthesis by binding to the D-ala-D-ala terminus of the tetrapeptide chain, which also results in the inhibition of PBPs [44]. Bacitracin inactivates the membrane carrier, bactoprenol, responsible for the transport of peptidoglycan building blocks from the cytoplasm to the cell wall [47].

### 3.2. Inhibition of Protein Synthesis

Protein synthesis involves mRNA, tRNA, ribosomes, and other cytoplasmic factors and consists of three steps: initiation, elongation, and termination. The bacterial ribosome has two subunits, the 50S and 30S, each composed of rRNA and proteins [46].

There are several classes of antimicrobials that act to inhibit protein synthesis by binding to the 30S subunit (aminoglycosides, tetracyclines, and glycylcyclines) or the 50S subunit (macrolides, chloramphenicol, oxazolidinones, lincosamides, and streptogramin) [44,48,49,50]. Aminoglycosides act by binding with high affinity to the 16S rRNA of the 30S subunit. Thus, codons are misread when aminoacyl tRNA is delivered, resulting in erroneous protein synthesis. Consequently, the wrong amino acids are compiled into a polypeptide that is released, leading to apoptosis [44]. Tetracyclines, on the other hand, act through passive diffusion in the cell membrane by porin channels and reversibly bind to the 30S subunit, resulting in blocking the binding of the tRNA to the mRNA-ribosome complex [44]. Glycylcyclines are an antimicrobial class developed to overcome the mechanisms of resistance to tetracycline (ribosomal protection and efflux pumps). They bind to the 30S subunit with five times more affinity, inhibiting protein synthesis. On the other hand, glycylcyclines are not recognized by the tetracycline efflux transporter, exhibiting significant antibacterial activity [50].

Macrolides and oxazolidinones bind to the 23S rRNA of the 50S subunit and inhibit the process of translocation or transpeptidation of protein synthesis, inducing a premature separation of incomplete peptide chains. Chloramphenicol crosses the cell membrane and reversibly binds to the L16 protein of the 50S subunit, thus inhibiting the formation of peptide bonds and preventing the elongation of peptide chains [44].

Additionally, nitrofurans are bacteriostatic antimicrobials whose multiple mechanisms of action are not fully understood [51]. They inhibit the synthesis of proteins, DNA, and RNA [52]. Moreover, their wide mechanisms of action may explain the lack of acquired bacterial resistance to nitrofurans [51,52].

Very recently, streptothricin F has been revisited as a bactericidal antimicrobial effective against highly drug-resistant Gram-negative bacteria, namely carbapenem-resistant Enterobacterales (CRE), *Acinetobacter baumannii*, and *Brucella abortus*, as well as *Mycobacterium tuberculosis*. Streptothricin is a natural product mixture, currently referred to as nourseothricin. Its therapeutic use was abandoned due to its induced reversible kidney toxicity; however, new cytotoxic studies have shown that streptothricin F exhibits at least 10-fold lower toxicity than streptothricin D and nourseothricin, both in vitro and in vivo. Moreover, streptothricin F has an alternative and unique mechanism of action, interacting with the 30S subunit of the 70S ribosome [53].

### 3.3. Inhibition of Nucleic Acid Synthesis

Examples of antimicrobials that inhibit nucleic acid synthesis are ansamycins (e.g., rifamycin and rifampicin), fluoroquinolones, and nitroimidazoles (e.g., metronidazole) [44,46,54].

Ansamycins bind to the β-subunit of RNA polymerase, blocking RNA elongation and inhibiting RNA synthesis [44]. Fluoroquinolones act by inhibiting DNA gyrase and other topoisomerases, interfering with DNA replication [44,46]. Nitroimidazoles inhibit nucleic acid synthesis by forming nitroso radicals, which disrupt DNA. This class of antimicrobials is only effective against anaerobic bacteria, whose ferredoxin reduces them to active radicals [54].

### 3.4. Inhibition of Metabolic Pathways

Nitrogenous bases (purines and pyrimidines), formed through the folic acid pathway, are necessary for the synthesis of nucleic acids. This process is initiated with para-aminobenzoic acid (PABA), which is catalysed in dihydroflolic acid and subsequently in tetrahydrofolic acid, which is later used to synthesize nitrogenous bases [46].

Antimicrobials that inhibit folic acid synthesis are sulphonamides and trimethoprim [44]. Sulphonamides are structural analogues of PABA, competitively inhibiting the enzymatic conversion that leads to the production of dihydroflolic acid [44]. As for trimethoprim, it reversely inhibits the formation of tetrahydrofolic acid [44]. Used separately, trimethoprim and sulphonamides are bacteriostatic; however, combined, they seem to have a bactericidal effect [44].

### 3.5. Inhibition of Cell Membrane Function

Only a small class of antimicrobials act by inhibiting cell membrane function, the polymyxins. This class of lipopeptides consists of lipophilic detergent-type antimicrobials, which lyse cell membranes by destroying the lipopolysaccharide (LPS) layer [44].

## 4. Antimicrobial Resistance

Bacterial antimicrobial resistance may be natural or acquired. Natural resistance is either innate when constitutively expressed, or mediated if triggered by an antibiotic treatment. On the other hand, acquired resistance occurs through DNA mutation or via the transfer of genetic material between bacteria [44].

Bacteria can acquire antimicrobial resistance through genetic mutation, namely spontaneous mutation, hypermutation, and adaptive mutation. Spontaneous mutation can be driven by several factors, mainly errors in DNA replication, such as transitions, transversions, insertions and deletions, which are transmitted to the progeny. Hypermutation plays a crucial role in the evolution of antimicrobial resistance. Hypermutation is regulated by the SOS-inducible DNA polymerase IV. This mutation occurs in bacteria called hypermutators, as they have a greater affinity to undergo spontaneous mutations due to defects or repairs in DNA, or errors in the avoidance system. Therefore, hypermutators can quickly adapt to antimicrobials. Finally, adaptive mutation arises in non-diving bacteria, upon non-lethal selective pressure, such as nutrient conditions, or sub-inhibitory antimicrobial concentrations. This type of mutation is transient and can be reverted to the original condition in the absence of the pressure factor [45].

**Figure 2:**
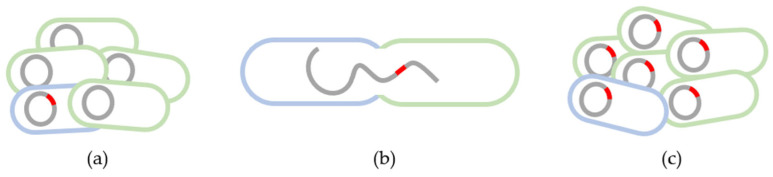
Horizontal gene transfer through conjugation. (**a**) Microbial community with antimicrobial susceptible bacteria (green) and antimicrobial resistant bacteria (blue); (**b**) Fusion between resistant and susceptible bacteria, allowing the transfer of genetic information through conjugation pili; (**c**) Microbial community with antimicrobial resistant bacteria.

Antimicrobial resistance genes can be acquired by horizontal gene transfer between bacteria, either by conjugation, transformation, or transduction [21,55,56,57]. Conjugation (Figure 2) is the transient fusion between two bacteria, where the transfer of genetic material takes place from the donor to the recipient through conjugation pili. Transformation is the uptake of free genetic material, released by a donor bacterium, by a recipient bacterium. Finally, transduction is the transfer of resistant genes mediated by bacteriophages [21,55,57]. Among AMR gene transfer mechanisms, conjugation has been shown to play an important role in the transmission and dissemination of AMR in food [18].

The persistent use of antimicrobials, as well as misuse and self-medication, leads to the abovementioned acquired AMR. Moreover, the appearance of MDR, bacteria resistant to three or more antimicrobial classes, is a critical public health problem [2,22,33,58]. The treatment of infections caused by MDR bacteria poses a relevant clinical challenge since the increase in AMR leads to higher rates of therapeutic failures, relapses, longer hospitalizations, and worse clinical outcomes [3].

The increase in AMR triggers the need for surveillance of bacteria resistant to antimicrobials, which has been carried out in public health and food safety laboratories through Whole Genome Sequencing (WGS), enabling the description of the full AMR profile [21,59].

**Figure 3:**
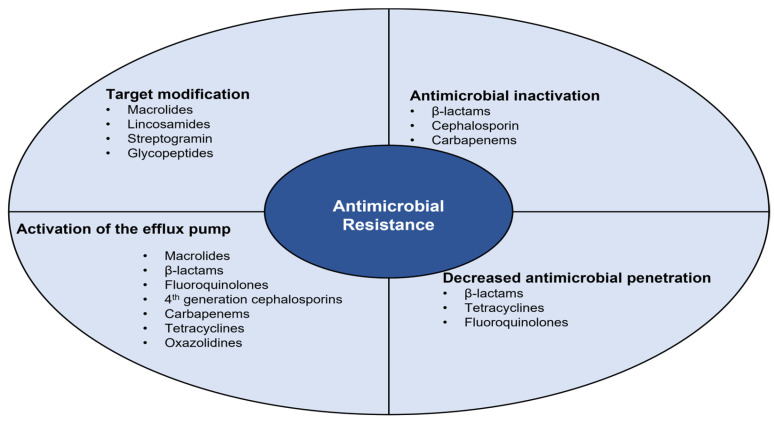
Biochemical mechanisms involved in the different bacterial resistance processes.

Bacterial resistance processes are divided into four biochemical mechanisms, which are highlighted in Figure 3 [44,45].

### 4.1. Antimicrobial Inactivation

Inactivation of the antimicrobial molecule occurs through the action of enzymes produced by resistant bacteria, such as β-lactamases and aminoglycoside-modifying enzymes [44,45,60]. Enzymes act on the antimicrobial molecule through hydrolysis, group transfer, or redox process. Hydrolysis is the process of destruction of the β-lactam ring of penicillin, cephalosporins, and carbapenems by β-lactamase-producing bacteria. Acyltransferases, phosphotransferases, and thioltransferases are examples of enzymes involved in hydrolysis, causing the destruction of the β-lactam ring and inhibiting the antimicrobial molecule binding to PBPs [44,45,60]. Group transfer, namely phosphoryl, acetyl, or adenyl group transfer to the antimicrobial active molecule, is considered the most effective mechanism of antimicrobial inactivation. An example of group transfer is acetylation on aminoglycosides, where enzymes alter hydroxyl or amino groups covalently, rendering antimicrobials inactive [44]. Finally, the redox process is the least studied mechanism, where antimicrobials are inactivated by oxidation or reduction [45].

### 4.2. Decreased Antimicrobial Penetration

Decreased antimicrobial penetration occurs through decreased cell wall permeability [44,45]. Gram-negative bacteria are intrinsically less permeable to certain antimicrobials than Gram-positive bacteria due to the large layer of LPS in the outer membrane of the cell wall that creates a permeability shield [44]. Hydrophilic molecules may penetrate the Gram-negative cell wall through porin proteins [45]. However, high-molecular-weight hydrophilic molecules, such as vancomycin, cannot pass through porins and are thus ineffective against Gram-negative bacteria [44].

Some bacteria are able to downregulate the expression of porins or even replace them with non-selective channels, decreasing the cell wall permeability and becoming thus resistant to some antimicrobials [44]. Hydrophilic molecules, such as β-lactams, tetracyclines, and some fluoroquinolones, are greatly affected by changes in the permeability of the outer membrane [44].

### 4.3. Activation of the Efflux Pump

The efflux system consists of energy-dependent membrane transport systems that pump a wide range of molecules [60]. In this transport system, there are efflux pumps, which are transport proteins that are located mostly in the bacterial cytoplasmic membrane [45,60]. These proteins transport nutrients and excrete cellular toxic compounds through the proton matrix force [45].

Efflux pumps can be specific to a particular antimicrobial or multi-resistant efflux pumps capable of excreting various antimicrobials [44,45,60]. The main families of efflux pumps are ATP-binding cassettes (ABC), small multidrug resistance (SMR), multidrug and toxic component extrusion (MATE), resistance-nodulation cell division (RND), and large facilitator superfamily (MFS) [44,60]. This mechanism confers resistance to macrolides, β-lactams, fluoroquinolones, 4th generation cephalosporins, carbapenems, tetracyclines, and oxazolidines [44,45].

### 4.4. Target Modification

The modification of the antimicrobial target is one of the most common resistance mechanisms. For β-lactams, changes may occur either in the composition or the amount of PBPs. Thus, the amount of antimicrobial that can bind to the target is affected by the change in the number of PBPs, while a structural modification decreases or completely prevents the binding of the molecules [44,60]. Another method is the production of alternative proteins that adopt the role of the bacterium’s native protein, resulting in antimicrobial resistance [45]. Moreover, modification of ribosomes or the peptidoglycan precursor can also occur. Ribosome modification consists of ribosome methylation, commonly mediated by *erm* gene products, which can be constitutive or inducible. This modification results in resistance to macrolides, lincosamides, and streptogramin B [60]. Regarding the modification of the peptidoglycan precursor, in the case of resistance to glycopeptides, it occurs through an amino acid substitution. The change occurs at the end of the D-alanyl-D-alanine dipeptide that is found at the terminals of the tetrapeptide [60].

## 5. Antimicrobial Resistance and Farm-to-Fork Transmission

Antimicrobial resistance in foods is considered a food safety issue but also a relevant public health problem. Furthermore, awareness of the prevalence of foodborne pathogenic bacterial strains resistant to antimicrobials is of the utmost importance [21,33].

The presence of bacteria resistant to antimicrobials in foods of animal origin has increased dramatically in recent years [33,61]. Moreover, bacteria have the ability to evolve and gain resistance to new antimicrobials [22,33,61]. Therefore, humans are highly exposed to AMR bacteria through food consumption [21,22,61,62], mainly due to the use of antimicrobials in the livestock sector [21,33,61,62]. Thus, the food chain has a high impact on the transmission of AMR, as food is not sterile and usually gets microbiological contamination via cross-contamination or recontamination throughout manufacturing. So, the food chain is considered a driver for the transmission of AMR bacteria [22,61,63,64].

The transfer of AMR bacteria from food products to humans occurs by consumption, followed by the horizontal transfer of resistance genes in the human gut [21,22,65]. Recently, several studies have studied the microbiome along the production chain to assess the AMR genes present in food samples [61,66]. These microbiome studies may contribute to the production of safer meat and meat products within the framework of One Health [66].

Therefore, several policy objectives have been considered to reduce antimicrobials in foods, such as a 50% reduction in the sales of antimicrobials for farmed animals and in aquaculture until 2030, aligned with the Farm-to-Fork Strategy of the European Green Deal [67]. Nevertheless, this may not be enough to effectively control AMR [68].

### 5.1. The Role of Meat in the Transmission of Antimicrobial Resistance

Meat and meat products are an important source of protein, vitamins, and minerals in the human diet and, in some countries, play an important role in gastronomic culture [69,70,71,72]. From the nutritional point of view, meat is considered a valuable source of protein, due to its amino acid composition, along with the presence of iron, zinc, and vitamins B12 and D, as well as other micronutrients [69,71]. The consumption of meat has been increasingly growing and is expected to reach between 460 and 570 million tons per year by 2050 [73,74].

Along with the increase in meat consumption, the demand for meat products has also grown, mainly due to their sensory properties and the opportunity to use parts of the carcass that cannot be used for fresh consumption [69,72]. Another advantage of meat products is their extended shelf-life. Meanwhile, meat products are also a vehicle for microorganisms, with either a beneficial, neutral, or harmful effect on health [72].

Within the animal industry, the rearing and consumption of pork meat have grown enormously in recent years, mainly because pork is a high-quality, low-cost animal protein [70]. However, pigs are considered one of the biggest reservoirs of AMR [65,75,76,77], mainly due to the inappropriate use of antimicrobials. In some countries, the administration of antimicrobials to promote animal growth is still allowed [78,79,80]. Excessive use of antimicrobials applies a selective pressure that leads to the development of antimicrobial-resistant bacterial populations, which may later be transmitted to humans [79].

Every step of the pig value chain, whether feeding, slaughtering, or processing, has the potential to affect human and animal health [38]. Moreover, there are two main sources of contamination in a pig slaughterhouse: the microorganisms carried on the pig’s skin and those from the evisceration step [81]. Mitigation measures along the food production chain may include enhanced disinfection procedures in the above-mentioned contamination-source areas in order to reduce the risk to food safety and consumer health due to the spread of antibiotic and virulence determinants to end products and the environment [81].

Hypervirulent clonal complexes (CCs) of *Listeria monocytogenes* were found in pig tonsils, showing the potential risk of pigs as source of isolates causing human listeriosis [82]. Moreover, a broad distribution of CC was observed along the whole pig production chain, suggesting multiple sources of entry [82].

Food contamination is the main cause of foodborne illnesses in both developed and developing countries [31]. Moreover, farm-to-fork AMR transmission is an additional food safety concern [83]. Considering the estimation that AMR will cause about 10 million deaths per year and cost US$100 trillion by 2050 [31,84,85], it is mostly relevant to control food contamination throughout the whole value chain.

Due to the impact of AMR and the fact that meat and meat products are highly consumed, there has been an increase in studies to evaluate the quality and safety of this type of food [72,86,87,88], including the search for antimicrobial-resistant pathogens in food (for example, *Staphylococcus aureus*, *Escherichia coli*, and *Salmonella* spp.), the associated antimicrobial resistance genes (Table 1), and the possibility of transmission to humans through consumption [86,89,90,91,92,93,94,95].

The microbiota of meat and meat products includes not only foodborne pathogens but also spoilage and technological microorganisms, which may all be responsible for farm-to-fork transmission of AMR [4,83]. For example, technological microbiota like coagulase-negative staphylococci can harbour antimicrobial resistance genes by acquiring them from other bacterial genera, normally pathogenic bacteria through horizontal gene transfer [105,106].

Additionally, there are studies that confirm the hypothesis of foodborne bacteria transferring antimicrobial resistance genes to the human gut microbiota [89,90,106,107,108,109]. Cao et al. (2022) worked with samples from 21 volunteers and pig and poultry carcasses and detected the presence of antimicrobial resistance genes (AMRGs) both in humans and food animals, conferring resistance to several antimicrobial classes: vancomycin, tetracycline and macrolides [90]. Moreover, this study showed that approximately 40% of AMRGs were shared between humans and pork, and 24.7% were shared between humans and poultry [90]. Bouchami et al. (2020) studied pigs, slaughter workers, and food contact surfaces. *Staphylococcus aureus* was selectively isolated from 41% of samples, 55% of which harboured the SCC*mec* type V cassette (methicillin-resistant *S. aureus—*MRSA), conferring resistance mainly to β-lactams, tetracycline, clindamycin, erythromycin, gentamicin and chloramphenicol [89]. These authors compared isolates from different sources and suggested the dissemination of MRSA from the pig production chain to humans. Lawal et al. (2021) evaluated *Staphylococcus saprophyticus* from human and slaughterhouse samples (equipment, pork meat, workers’ hands, and pigs’ rectum) [106]. The authors found AMRGs conferring resistance mainly to biocides (*qaac*) and trimethoprim (*dfr*G), both in foodborne and human isolates. *L. monocytogenes* was isolated from 12.5% of ready-to-eat meat-based products (RTEMBP), and 20% of the samples were considered MDR (resistant to gentamicin, meropenem, benzylpenicillin, quinupristin-dalfopristin, rifampin, sulphamethoxazole-trimethoprim, and tetracycline) [107]. The authors also detected a high similarity between RTEMBP and human clinical isolates.

These facts highlight the suitability of the One Health approach to control the AMR transmission process between food animals and humans.

### 5.2. Antimicrobial Resistance in Staphylococcus aureus

*Staphylococcus aureus* is an opportunistic, Gram-positive, round-shaped, facultative anaerobic pathogen that can often be found in the natural microbiota of both the nose and skin [25,110,111,112,113]. *S. aureus* is responsible for several life-threatening infections, such as endocarditis, toxic shock syndrome, and osteomyelitis [25,114,115]. *S. aureus* can also be found in foods (raw or ready-to-eat foods) due to contamination through the handling process, food-producing animals, and food contact surfaces [25,111,113]. High *S. aureus* load in food may cause food poisoning [25,111,113,115]. Moreover, *S. aureus* has been considered one of the most relevant microbiological hazards in meat and meat products because of their strong evidence association with foodborne outbreaks [116].

The excessive use of antimicrobials in the livestock sector led to the emergence of multi-resistant *S. aureus* in the food [30]. The most studied *S. aureus* is methicillin-resistant *S. aureus* (MRSA), because of the few effective treatments against infections with these strains [114].

The resistance to antimicrobials by *S. aureus* is due to mechanisms of intrinsic resistance, resistance mutations, or the acquisition of resistance mechanisms. Therefore, it is necessary to characterize the acquired resistance mechanisms through whole genome sequencing [111,117]. Methicillin-resistant *S. aureus* is considered multidrug-resistant bacteria, as resistance to almost all β-lactams, vancomycin, and fluoroquinolones has been reported [114,118]. MRSA resistance is due to horizontal transfer of genes and mobile genetic elements, such as the mobile staphylococcal cassette chromosome (SCC*mec*) that harbours the *mec*A, *mec*B, and *mec*C genes [25,111,119]. Regarding vancomycin resistance, it is conferred by the horizontal transfer of the *van*A gene from *Enterococcus* spp. to *S. aureus* [120].

Evidence of foodborne transmission of MRSA has been reported by several authors [121,122]. Similarly, Bonardi et al. (2022) found a genetic relationship between swine and human isolates, although no direct epidemiological link was demonstrated [123].

### 5.3. Antimicrobial Resistance in ESBL-Producing Enterobacteriaceae

*Enterobacteriaceae* is a family of Gram-negative bacilli, facultative anaerobes, responsible for various community-acquired and nosocomial infections, such as urinary, lower respiratory tract and bloodstream infections [124,125,126]. The natural habitat of enterobacteria is the gut of humans and animals [28]. In recent years, *Escherichia coli* and *Klebsiella pneumoniae*, both ESBL-producing enterobacteria, have been the main species associated with nosocomial infections [28,124,127,128]. ESBL-producing enterobacteria are a group of bacteria consisting of *K. pneumoniae*, *E. coli*, *Enterobacter* spp, *Proteus* spp., *Serratia* spp., *Providencia* spp., *Salmonella* spp., and *Morganella morganii*, which are resistant to a wide range of β-lactams [28]. Due to their MDR resistance profile, ESBL enterobacteria are considered a critical priority in the WHO “List of Priority Pathogens” [129], which represents a public health problem, also being detected in the livestock and food sectors [28,130].

The extensive resistance of enterobacteria to β-lactams is due to the widespread use of these antimicrobials. ESBL are bacterial enzymes that confer resistance to broad-spectrum penicillin, among other β-lactams, like third-generation cephalosporines [28,125,131]. A specific ESBL produced by enterobacteria is *Amp*C β-lactamase, which is capable of hydrolysing penicillins, 1st to 3rd generation cephalosporins, cephamycins, and beta-lactamase inhibitors [132,133,134,135]. Additionally, metallo-beta-lactamases confer resistance to carbapenems [134,135].

Furthermore, enterobacteria often harbour *mcr* genes that confer resistance to colistin [130,132,136]. Recently, colistin-resistant enterobacteria have been reported on a large scale [124,137]. This resistance is due to a lower binding affinity for colistin, through the modification of the lipid A component of LPS. *mcr* genes are found in plasmids, accelerating the transfer of resistance between bacterial strains [124].

Identical strains of ESBL-*E. coli* were isolated from both healthy humans and swine [138]. Moreover, common transposable elements were found in ESBL-*E. coli* isolates from human and non-human sources [139].

### 5.4. Antimicrobial Resistance in Vancomycin-Resistant Enterococcus spp.

Lactic acid bacteria (LAB) are Gram-positive cocci or bacilli, non-spore-forming, anaerobic, catalase-negative, and able to ferment glucose, resulting in the production of lactic acid, CO_2_, and ethanol [140,141]. Although most LABs are beneficial, some species are opportunistic pathogens for animals and humans, as is the case of some enterococci commonly found in the gastrointestinal tract [29,140,142]. Within LAB, *E. faecium* and *E. faecalis* are the most problematic species, responsible for nosocomial infections such as bloodstream, urinary tract, endocardium, and skin infections [26,141,143,144,145]. In recent years, infections with antimicrobial-resistant enterococci have been reported due to their intrinsic resistance to vancomycin and penicillin and both intrinsic and acquired resistance to aminoglycosides and macrolides [26,146,147,148]. Furthermore, a small percentage of linezolid-resistant enterococci, as well as enterococci with a low susceptibility to both linezolid and tedizolid, have been reported [26,147,148,149].

However, vancomycin-resistant enterococci are the main opportunistic pathogens, being classified as high-priority pathogens by the WHO [129]. They may be found in foods due to their ability to adapt to various environmental conditions, such as the production and storage environments for ready-to-eat foods [29,150]. Bearing in mind that enterococci have very plastic genomes capable of acquiring and transferring resistance to antimicrobials, enterococcal infections are very difficult to treat because enterococci easily become multidrug-resistant [142]. Enterococci resistance to vancomycin is due to the acquisition of *van* genes. While *van*A is widely used in the identification of mobile genetic elements, *van*C1, *van*C2, and *van*C3 genes are responsible for the intrinsic resistance of enterococci [147,150]. When vancomycin-resistant enterococci also exhibit resistance to ampicillin, treatment of infections is usually limited to the use of last-resort antimicrobials such as linezolid, tigecycline, and daptomycin [146,151]. However, enterococci resistant to oxazolidinones (linezolid, tedizolid) have arisen, which results from the acquisition of transferable plasmid genes, namely *cfr*, *cfr* (B), *cfr* (C), and *optr*A, mutations in the 23S rRNA genes, and mutations in the ribosomal proteins L3 and L4 genes [146,149].

Identical strains of *Enterococcus faecalis* resistant to gentamicin have been found in patients and pigs in Denmark [152].

### 5.5. Antimicrobial Resistance in ESKAPE Bacteria

ESKAPE bacteria are considered one of the greatest dangers in modern medicine, because they are MDR bacteria, often causing nosocomial infections [27], which are one of the main causes of morbidity and mortality across the world [2,153]. All bacteria that belong to this group are opportunistic pathogens, showing several antimicrobial resistance mechanisms, such as target modification, enzymatic inactivation, and mechanical protection (biofilm formation) [27,154]. ESKAPE bacteria belong to the list of WHO pathogens, Priority 1 (Critical antibiotic resistance), and Priority 2 (High antibiotic resistance) levels [129]. Gram-negative ESKAPE bacteria belong to Priority 1 (*Klebsiella pneumoniae*, *Acinetobacter baumannii*, *Pseudomonas aeruginosa*, and *Enterobacter* spp.), while Gram-positive ESKAPE bacteria are Priority 2 (*Enterococcus faecium* and *Staphylococcus aureus*) [153]. Among the resistance mechanisms of ESKAPE bacteria, biofilm formation has been the focus of greatest concern, with biofilm acting as a physical barrier to host immune mechanisms and antimicrobial molecules. In fact, biofilms can even protect antimicrobial-tolerant bacteria [2].

*Enterococcus faecium* and *Staphylococcus aureus* are two clinically relevant Gram-positive bacteria, frequently responsible for nosocomial infections [27].

*Klebsiella pneumoniae* is a Gram-negative bacterium of the *Enterobacteriaceae* family. These are encapsulated rod-shaped, facultative anaerobes found in the gastrointestinal tract, responsible for several infections, such as urinary infections and pneumonia. The resistance of *K. pneumoniae* to antimicrobials is due to the production of extended-spectrum β-lactamases (ESBL), which putatively confer resistance to β-lactams, cephalosporines, monobactams and carbapenems [27,153,155].

*Acinetobacter baumannii* is a Gram-negative coccobacillus, which is strictly aerobic and non-fermentative. It is frequently found in hospital environments, causing bloodstream infections, among others. This bacterium has a high ability to survive on surfaces due to biofilm production, produces ESBL, its genome evolves rapidly; and it can acquire AMR genes under selective pressure [27,153,155].

*Pseudomonas aeruginosa* is a Gram-negative rod-shaped, strictly aerobic, encapsulated bacterium considered an opportunistic pathogen. It can cause sepsis, pneumonia, and other difficult-to-treat infections. Its resistance to antimicrobials is due to the acquisition of mobile resistance genes, biofilm formation, and expression of porins and efflux pumps, resulting in resistance to colistin, chloramphenicol, tetracycline, β-lactams, rifampin, and trimethoprim-sulfamethoxazole [27,153,155].

*Enterobacter* is a Gram-negative bacillus of the *Enterobacteriaceae* family, a facultative anaerobe, belonging to the human microbiota. It is an opportunistic pathogen that causes infections, such as pneumonia, sepsis, and urinary tract infections, among others. *Enterobacter* is intrinsically resistant to ampicillin, amoxicillin, first-generation cephalosporins, and cefoxitin due to the presence of a constitutive AmpC β-lactamase [156]. Since they produce ESBL and carbapenemases, they further harbour various resistance *bla* genes (*bla*NDM, *bla*OXA, *bla*KPC, *bla*VIM, *bla*CTX-M, *bla*IMP, and *bla*TEM) [27,153,155].

## 6. Conclusions

Despite the guidelines that have been implemented worldwide, and especially in Europe, within the scope of antimicrobial stewardship, and the efforts made by professionals involved in human and animal health nowadays, AMR is still a recurrent global problem responsible for high morbidity rates, leading to thousands of deaths each year.

Besides being a problem for causing foodborne infections or intoxications, foodborne bacteria can also carry antimicrobial resistance genes. Specifically, in pork meat products, antimicrobial resistance genes have been detected for ampicillin, chloramphenicol, clindamycin, gentamycin, kanamycin, nitrofurantoin, quinolone, streptomycin, tetracycline, trimethoprim, and tylosin. Moreover, in the human digestive tract, transfer of resistance genes to indigenous gut bacteria may occur. Additionally, identical isolates and highly similar antimicrobial resistance genes were detected in meat and meat products, other ready-to-eat meat-based food, and human clinical isolates.

Therefore, AMR foodborne (from foods to humans) transmission, or “farm-to-fork” transmission, has been reported and should be of the utmost concern, particularly in the case of pork meat and meat products. Furthermore, from a One Health perspective, different disciplines are necessary and should be integrated to control the problem of AMR globally.

## Figures and Tables

**Table 1:** Antimicrobial resistance genes identified in different types of meat and meat products.

Type of Food	Microbiota	Antimicrobial Resistance Genes	Reference
Raw poultry, pork and beef	*Enterococcus* spp.	Vancomycin: *van*A, *van*B and *van*C1,2,3	[96]
		Tetracycline: *tet*M, *tet*L	
		Erythromycin: *erm*A and *erm*B	
		Quinupristin-dalfopristin: *vat*[D] and *vat*[E]	
Retail poultry meat	*Escherichia coli*	β-lactam: *bla*TEM, *bla*SHV, *bla*CMY-2 and *bla*CTX-M	[86]
		Sulphamethoxazole: *sul*2	
		Tetracycline: *tet*A and *tet*B	
		Chloramphenicol: *cml*A	
		Aminoglycoside: *aph*A1 and *aad*A	
		Trimethoprim: *dfr*A1	
Bull-cooked meat products	*Enterobacter* spp. *Escherichia coli* *Citrobacter* spp. *Pseudomonas* spp.	β-lactam: *bla*TEM-1 and *bla*CTX-M-14	[88]
		Gentamicin: *aac(3)*-*IIa*	
		Streptomycin: *str*A and *str*B	
		Quinolone: *qnr*B and *qnr*S	
		Sulphamethoxazole: *sul*1, *sul*2 and *sul*3	
		Chloramphenicol: *cat*1 and *cat*3	
		Tetracycline: *tet*M. *tet*A and *tet*B	
Animal-based products (ready-to-eat food)	*Staphylococcus saprophyticus* *Staphylococcus sciuri* *Staphylococcus xylosus*.	Oxacillin: *mec*A	[95]
		β-lactam: *bla*Z	
		Tetracycline: *tet*K	
		Erythromycin: *msr*A, *msr*B, *erm*A	
		Gentamycin: *aac*A-*aph*D	
		Fusidic acid: *fus*D	
		Trimethoprim/sulfamethoxazole: *dfr*G	
Chicken meat	*Salmonella* Albany *Salmonella* Virchow *Salmonella* Enteritidis *Salmonella* Infantis	β-lactam: *bla*CTZ-M-15, *bla*CTX-M-79 and *bla*CMY-2	[93]
		Tetracycline: *tet*A and *tet*B	
		Sulfonamide: *sul*1 and *sul*2	
		Chloramphenicol: *cat*A1 and *cml*A	
Retail meat (pork, chicken and duck)	*Salmonella* Enteritidis *Salmonella* Typhimurium *Salmonella* Typhi *Salmonella* Goldcoast *Salmonella* Ouakam *Salmonella* Paratyphi	Tetracycline: *tet*A	[94]
		β-lactam: *bla*TEM	
		Aminoglycoside: *aad*A1 and *aad*A2	
		Sulfonamide: *sul*1 and *sul*2	
Dry fermented Italian salami	*Enterococcus faecium* UC7251	Ampicillin: *pbp*5-S_1_/R_20_	[97]
		Gentamycin: *aac(6′)-li*	
		Kanamycin: *aph(3′)-lll*	
		Streptomycin: *aad*6 and *aad*E	
		Erythromycin: *erm*B, *mrs*C and *sat*4	
		Clindamycin: *erm*B, *lnu*B and *lsa*E	
		Tylosine: *erm*B	
		Tetracycline: *tet*L and *tet*M	
Traditional pork dry sausages	*Salmonella* Enteritidis *Salmonella* Typhi *Salmonella* Typhimurium	Quinolone: *gyr*A and *parC*	[91]
		Chloramphenicol: *cat*A1	
		Trimethoprim: *drf*A	
		Tetracycline: *tet*A and *tet*B	
		Nitrofurantoin: *nfs*A and *nfs*B	
		Ampicillin: *bla*TEM	
Chicken meat	*Escherichia coli* isolate 1108	β-lactam: *bla*NDM-1, *bla*TEM-1, *bla*CTZX-M-64 and *bla*CMY-2	[92]
		Bleomycin: *ble*MBL	
		Sulfonamide: *sul*1 and *sul*2	
		Tetracycline: *tet*A and *tet*R	
		Aminoglycosides: *str*A	
		Quinolone: *oqx*A and *oqx*B	
		Phenicol: *floR*	
		Streptomycin: *aad*A2	
		Trimethoprim: *dfr*A12	
Retail meat (chicken and pork)	*Salmonella* Kentucky *Salmonella* Indiana *Salmonella* Derby *Salmonella* Typhimurium *Salmonella* Litchfield *Salmonella* Schwarzengrun	β-lactam: *bla*CTX-M-55, *bla*TEM-206, *bla*TEM-214, *bla*OXA-1, *bla*CTX-M-123, *bla*TEM-1, *bla*CTX-M-64 and *bla*CTX-M-15	[98]
Naturally fermented smoked pork	*Staphylococcus carnosus* *Lactobacillus plantarum* *Labctobacillus brevis* *Lactobacillus sakei* *Weissella confusa* *Weissella cibaria*	Tetracycline: *tet*O and *tet*M Erythromycin: *ere*A Chloramphenicol: *cat*A Streptomycin: *str*A and *str*B	[99]
Pork meat	*Aeromonas aquariorum* *Aeromonas hydrophila* *Aeromonas jandaei* *Aeromonas veronii* *Acinetobacter baumannii* *Acinetobacter bereziniae* *Acinetobacter johnsonii* *Acinetobacter septicus* *Acinetobacter ursingii* *Citrobacter* sp. *Citrobacter freundii* *Citrobacter murliniae* *Enterobacteriaceae* *Enterobacter* sp. *Enterobacter asburiae* *Enterobacter cloacae* *Enterobacter hormaechei* *Enterobacter ludwigii* *Escherichia coli* *Klebsiella* sp. *Klebsiella oxytoca* *Klebsiella terrigena* *Lactobacillus casei* *Leclercia* sp. *Lactococcus garvieae* *Lactococcus lactis* *Micrococcus caseolyticus* *Myroides phaeus* *Myroides marinus* *Myroides odoratimimus* *Oceanobacillus* *Pantoea* sp. *Pantoea dispersa* *Pantoea agglomerans* *Proteus penneri* *Providencia alcalifaciens* *Pseudomonas* sp. *Raoultella* sp. *Raoultella terrigena* *Serratia* sp. *Serratia marcescens* *Sphingobacterium* *Staphylococcus* sp. *Staphylococcus sciuri* *Staphylococcus epidermidis* *Vibrio cincinnatiensis* *Wautersiella falsenii* genomovar 1 *Kurthia* sp. *Bacillus* sp. *Morganella* sp. *Micrococcus caseolyticus* *Vagococcus* sp. *Raoultella ornithinolytica* *Comamonas* sp. *Budvicia* sp. *Aeromonas* sp. *Klebsiella* sp.	β-lactam: *bla*TEM, *bla*CTX-M, *bla*CMY-2 Tetracycline: *tet*A, *tet*C, *tet*E, *tet*K, *tet*L, *tet*M and *tet*S Sulfonamide: *sul*1 and *sul*2 Aminoglycoside: *aad*A and *aph*A-1 Chloramphenicol: *cml*A Macrolide: *erm*B Florfenicol: *flo*R	[100,101,102]
Raw and cooked pork	*Citrobacter freundii* *Serratia marcescens* *Escherichia coli*	β-lactams: *bla*TEM, blaCTX-M-1, *bla*SHV and *bla*CTX-M-9	[103]
Pork meat and pork meat preparations (cotechino, hamburger, sausages and Zuccotto of Bismantova)	*Salmonella* Derby *Salmonella* Typhimurium *Salmonella* Bredeney *Salmonella* London *Salmonella* Anatum *Salmonella* Agona *Salmonella* Virchow *Salmonella* Senftenberg *Salmonella* Livingstone *Salmonella* India *Salmonella* Heidelberg *Salmonella* Bovis-morbificans *Salmonella* Coeln	Ampicillin: *bla*PSE-1 Gentamicin: *ant (2″)-Ia* Sulfamethoxazole: *sul*1 Tetracycline: *tet*A, *tet*B, *tet*G and *mar*RAB	[104]

## Data Availability

Not applicable.
